# Comparison of Nonculture Blood-Based Tests for Diagnosing Invasive Aspergillosis in an Animal Model

**DOI:** 10.1128/JCM.03233-15

**Published:** 2016-03-25

**Authors:** P. Lewis White, Nathan P. Wiederhold, Juergen Loeffler, Laura K. Najvar, Willem Melchers, Monica Herrera, Stephane Bretagne, Brian Wickes, William R. Kirkpatrick, Rosemary A. Barnes, J. Peter Donnelly, Thomas F. Patterson

**Affiliations:** a PHW Microbiology, Cardiff, United Kingdom; b San Antonio Center for Medical Mycology, The University of Texas Health Science Center at San Antonio, San Antonio, Texas, USA; c Wuerzburg University, Wuerzburg, Germany; d Radboud University Nijmegen Medical Centre, Nijmegen, The Netherlands; e Hôpital Saint Louis, Paris, France; f Cardiff University, UHW, Cardiff, United Kingdom; g South Texas Veterans Health Care System, San Antonio, Texas, USA

## Abstract

The European Aspergillus PCR Initiative (EAPCRI) has provided recommendations for the PCR testing of whole blood (WB) and serum/plasma. It is important to test these recommended protocols on nonsimulated “*in vivo*” specimens before full clinical evaluation. The testing of an animal model of invasive aspergillosis (IA) overcomes the low incidence of disease and provides experimental design and control that is not possible in the clinical setting. Inadequate performance of the recommended protocols at this stage would require reassessment of methods before clinical trials are performed and utility assessed. The manuscript describes the performance of EAPCRI protocols in an animal model of invasive aspergillosis. Blood samples taken from a guinea pig model of IA were used for WB and serum PCR. Galactomannan and β-d-glucan detection were evaluated, with particular focus on the timing of positivity and on the interpretation of combination testing. The overall sensitivities for WB PCR, serum PCR, galactomannan, and β-d-glucan were 73%, 65%, 68%, and 46%, respectively. The corresponding specificities were 92%, 79%, 80%, and 100%, respectively. PCR provided the earliest indicator of IA, and increasing galactomannan and β-d-glucan values were indicators of disease progression. The combination of WB PCR with galactomannan and β-d-glucan proved optimal (area under the curve [AUC], 0.95), and IA was confidently diagnosed or excluded. The EAPRCI-recommended PCR protocols provide performance comparable to commercial antigen tests, and clinical trials are warranted. By combining multiple tests, IA can be excluded or confirmed, highlighting the need for a combined diagnostic strategy. However, this approach must be balanced against the practicality and cost of using multiple tests.

## INTRODUCTION

The diagnosis of invasive aspergillosis (IA) remains difficult, resulting in the widespread use of empirical therapy or posaconazole prophylaxis in high-risk patients. Definitive (proven) diagnosis of IA requires microscopic evidence of invasive fungal disease in tissue and/or culture of Aspergillus sp. from the diseased area (1). Most patients are diagnosed with probable or possible IA based on specific radiological signs with or without the detection of biomarkers (galactomannan [GM] or β-d-glucan [BDG]) or culture of Aspergillus (1).

Direct detection of Aspergillus in clinical specimens using molecular methods is yet to be included in criteria for defining invasive fungal disease (IFD), which is a result of limited standardization (1). The European Aspergillus PCR Initiative (EAPCRI) provided recommendations based on simulated samples to standardize procedures for whole blood (WB) and serum/plasma (2, 3). Following evaluation of analytical performance but before widespread multicenter clinical evaluation, it is important that these recommended protocols are used to test “nonsimulated” specimens to determine performance when detecting targets generated *in vivo*. Animal models have been used to evaluate the diagnosis and treatment of IA (4, 5). Animal models can overcome the relatively low incidence of disease and allow the application of experimental design and control, which is not possible in the clinical setting. Testing an animal model would support the initial clinical validation, and inadequate performance at this stage would highlight limitations that were necessary to overcome before large-scale clinical trials are performed (6–8).

In order to continue with the PCR standardization process, the EAPCRI formed an international collaboration with the NIH-founded Invasive Aspergillosis Animal Models (IAAM) group to evaluate the performance of the Aspergillus PCR protocols recommended by the EAPCRI when testing blood samples derived from a guinea pig model of IA. In addition to the performance of PCR, the detection of other circulating biomarkers, galactomannan (GM) and β-d-glucan (BDG), was also evaluated with particular focus given to the timing of positivity and the concept of combination testing, and the findings are described.

## MATERIALS AND METHODS

### Animal model of invasive aspergillosis.

Prior to inoculation with Aspergillus conidia, 43 male Hartley guinea pigs (Charles River Laboratory, Wilmington, MA) were immunosuppressed using intraperitoneal cyclophosphamide (Cytoxan; Mead Johnson, Princeton, NJ) and subcutaneous cortisone acetate (Sigma, St. Louis, MO) as previously described (4). In addition, animals received daily antibiotic prophylaxis with ceftazidime (50 mg/kg of body weight; GSK Beecham Pharmaceuticals, Philadelphia, PA) to prevent bacterial infection. All procedures were approved by the Institutional Animal Care and Use Committee at the University of Texas Health Science Center at San Antonio, and animals were maintained in accordance with the Association for the Assessment and Accreditation of Laboratory Animal Care (9).

On the day of inoculation, 30 immunosuppressed animals were placed individually in an acrylic chamber to permit inoculation with a nebulized suspension of Aspergillus fumigatus AF293 conidia (7 × 10^7^ conidia/ml) for 1 h with a flow rate of 100 kPa. Confirmation of the conidial delivery was accomplished by humanely killing one guinea pig to allow enumeration of the lung conidial load, which after 1 h of inoculation was 3.8 × 10^4^ CFU/g tissue. One hour, 5 days, 7 days, and 9 days after inoculation, infected animals (4, 13, 9, and 4, respectively, at each time point) were humanely killed, and whole blood samples were drawn by cardiac puncture. An additional 13 immunosuppressed animals served as uninfected controls that were potentially vulnerable to IA, and a further 13 immunocompetent animals served as uninfected IA invulnerable control animals. Control animals were euthanized at the corresponding time points, and disease was confirmed in a postmortem investigation of lung tissue (10). Throughout the study, animals were monitored daily for any obvious signs of illness or other signs of distress. Any animal found to be moribund before the end of the study was euthanized.

In keeping with EAPCRI recommendations, 3 ml whole blood (WB) (≈3 ml) was collected into EDTA Vacutainer tubes and was rocked for 5 min before being frozen at −80°C for PCR testing (2). WB without anticoagulant was fractionated by centrifugation, and the serum component was removed and stored at −80°C for GM, BDG, and PCR testing.

### Testing strategy.

GM and BDG testing was performed by the IAAM group. The EDTA WB samples for molecular testing were divided across four experienced EAPCRI centers (Public Health Wales Microbiology Cardiff; Radboud Medical Centre, Nijmegen; St. Louis Hospital, Paris; and University Hospital Wuerzburg). Serum samples were tested by one EAPCRI center (Public Health Wales Microbiology Cardiff). All methods were compliant with recommendations for the testing of serum and WB, providing optimal analytical performance (2, 3).

### GM testing.

The Bio-Rad Platelia Aspergillus enzyme immunoassay (EIA) kit was used to detect galactomannan using 300 μl of serum following manufacturer's instructions and a positivity index of 0.5.

### BDG testing.

BDG testing was performed using the Associates of Cape Cod Fungitell assay using 5 μl of serum and a positivity threshold of 80 pg/ml. Samples with a BDG concentration of between 60 and 79 pg/ml were considered indeterminate, and samples below 60 pg/ml were considered negative. The test was performed according to the manufacturer's instructions.

### Serum DNA extraction.

DNA was extracted from 0.5 ml of serum using the High Pure template DNA kit (Roche, Burgess Hill, United Kingdom). All DNA was eluted in 60 μl and generated excellent analytical performance when previously evaluated (3). Positive (healthy donor serum spiked with A. fumigatus genomic DNA) and negative (healthy donor serum) extraction controls were used to monitor extraction performance.

### Whole blood DNA extraction.

DNA was extracted from 3.0 ml of EDTA WB using the standard EAPCRI protocol as previously described; however, when erythrocytes persisted after 2 cycles of red cell lysis, an additional red cell lysis step was performed (2). After bead beating, two EAPCRI centers (Nijmegen and Paris) used the MagNA Pure LC large volume kit (Roche) and two centers (Wuerzburg and Cardiff) used the High Pure template DNA kit (Roche) for final DNA purification and elution. All DNA was eluted at a volume of <100 μl. The two methods generate excellent analytical performance as described previously (2). Positive extraction controls (healthy donor EDTA WB spiked with A. fumigatus conidia) and negative extraction controls (healthy donor EDTA WB) were included to monitor extraction performance.

### PCR amplification.

The EAPCRI centers performed Aspergillus-specific real-time PCR targeting the 28S rRNA unit (Cardiff and Nijmegen), the internal transcribed spacer 2 (ITS 2) region (Wuerzburg), and a combined approach using two PCRs targeting rRNA/mitochondrial DNA regions (Paris) (7, 11, 12). All methods have shown excellent analytical performance (2, 3). An internal control PCR was performed to monitor for inhibition as recommended (2, 3). All PCR testing was performed in duplicate, and, where possible, a third replicate was tested to resolve discrepant results.

### Statistical analysis.

To determine the clinical performances (sensitivity, specificity, likelihood ratios, overall accuracy, and diagnostic odds ratio) of the assays, 2 by 2 tables were constructed using the infected animals as true cases and uninfected animals as a control population. For all animals, only a single sample was tested by each assay; consequently, only a single positive was required to consider the animal positive by a specific test. For PCR testing, only individual samples that were confirmed by repeat testing (2/2 or 2/3 replicates positive) were considered PCR positive. For the proportionate values of the individual assays, 95% confidence intervals (95% CIs) and, when required for comparative purposes, *P* values (Fisher's exact test; *P* = 0.05) were generated to determine the significance of the differences between rates (13). For the individual quantitative assays (BDG and GM) and for combined assay strategies, receiver operator characteristic (ROC) curve analysis was performed, and the area under the curve (AUC) was calculated using GraphPad Prism 5 (GraphPad Software, La Jolla, CA, USA). For the ROC analysis of the combination of assays, the threshold used to determine positivity related to the number of positive assays required before confirming IA was explored.

## RESULTS

A total of four different assays were performed (GM and BDG on serum samples only and PCR testing of serum and whole blood) on the samples taken from the animal model. The individual performance of each assay is shown in Table 1.

**TABLE 1:** The diagnostic performance of individual assays compared with combination testing^*a*^

Assays^*b*^	Sensitivity (%)	Specificity (%)	Accuracy (%)	LR+^*c*^	LR−^*d*^	DOR^*e*^	ROC analysis, AUC (95% CI)
WB PCR^*f*^ (95% CI)	73 (54–86)	92 (76–98)	82.7	9.5	0.3	32.7	
Serum PCR (95% CI)	65 (46–81)	79 (60–91)	72.0	3.1	0.4	7.3	
GM (95% CI)	68 (47–84)	80 (61–91)	74.5	3.4	0.4	8.5	0.77 (0.62–0.91)
BDG (95% CI)	46 (27–65)	100 (86–100)	74.5	>455	0.6	>827.3	0.82 (0.70–0.95)
Serum/WB PCR	91/33	65/100	77/68	2.6/>330	0.1/0.7	18.6/>495	0.84 (0.72–0.96)
BDG/WB PCR	86/24	91/100	89/64	9.6/>240	0.2/0.8	48/>300	0.90 (0.79–1.00)
GM/WB PCR	100/33	78/96	89/66	4.5/8.3	<0.001/0.7	>4500/11.8	0.91 (0.81–1.00)
BDG/serum PCR	76/33	74/100	75/68	2.9/>330	0.3/0.7	9.7/>471	0.79 (0.66–0.93)
GM/serum PCR	90/43	61/96	75/70	2.3/10.8	0.2/0.6	11.5/18	0.82 (0.70–0.95)
GM/BDG	81/38	83/100	82/70	4.8/>380	0.2/0.6	24/>613	0.85 (0.73–0.97)
WB PCR/GM/BDG	100/67/14	78/96/100	89/82/59	4.5/16.8/>140	<0.001/0.3/0.9	>5000/57/>156	0.95 (0.88–1.00)
Serum PCR/GM/BDG	100/48/33	61/96/100	80/73/68	2.6/12/>330	<0.002/0.5/0.7	>1300/24/>471	0.88 (0.79–0.98)
WB/serum PCR/BDG	90/71/10	65/100/100	77/86/57	2.6/>710/>100	0.2/0.3/0.9	13/>2367/>111	0.90 (0.81–1.00)
WB/serum PCR/GM	100/81/14	57/91/100	77/86/59	2.3/9/>140	<0.002/0.2/0.9	>187/45/>156	0.92 (0.84–1.00)
All four assays	100/91/44/10	57/91/100/100	77/91/73/57	2.3/10.4/>429/>950	<0.001/0.1/0.6/0.9	>2300/104/>751/>1044	0.95 (0.90–1.00)

a For combined assay performance, the first figure represents the performance if one of the assays was required positive for the animal to be considered positive, the second figure if two assays were required positive, and so on. For example, for combined serum/WB PCR, if one of the assays was required positive then sensitivity and specificity of 91% and 65% were generated. If both were required positive, then sensitivity and specificity were 33% and 100%, respectively. For assays with 100% sensitivity or specificity, likelihood ratios and diagnostic odds ratio have been calculated using a value of 99.9% and are representative values used to replace ∞.

b GM, galactomannan Bio-Rad Aspergillus Ag test; BDG: β-d-glucan Associates of Cape Cod β-d-glucan test.

c LR+, likelihood ratio for a positive test result.

d LR−, likelihood ratio for a negative test result.

e DOR, diagnostic odds ratio.

f Excludes one case where the sample was inhibitory to PCR amplification.

### BDG performance.

**FIG 1:**
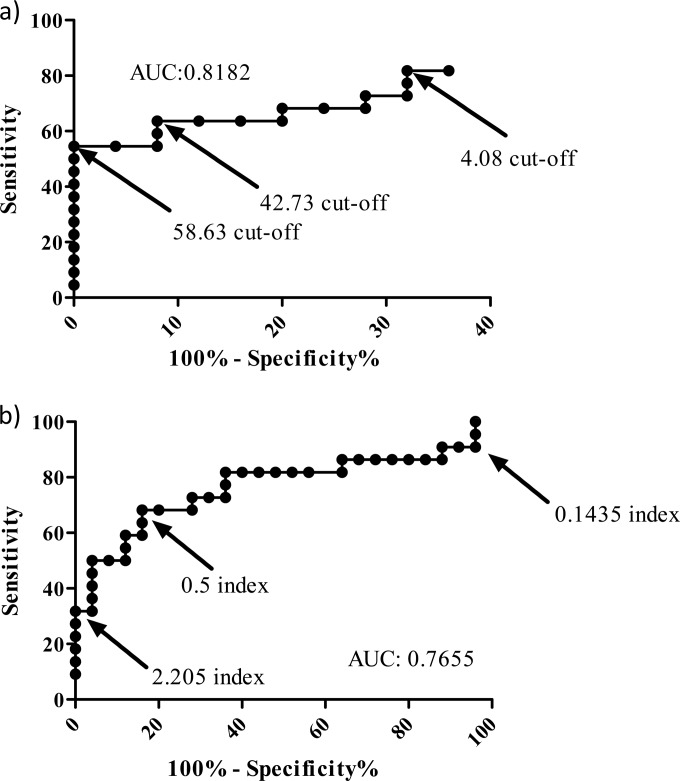
Receiver operator characteristic curves for β-d-glucan (a) and galactomannan enzyme-linked immunosorbent assay (ELISA) testing (b).

**FIG 2:**
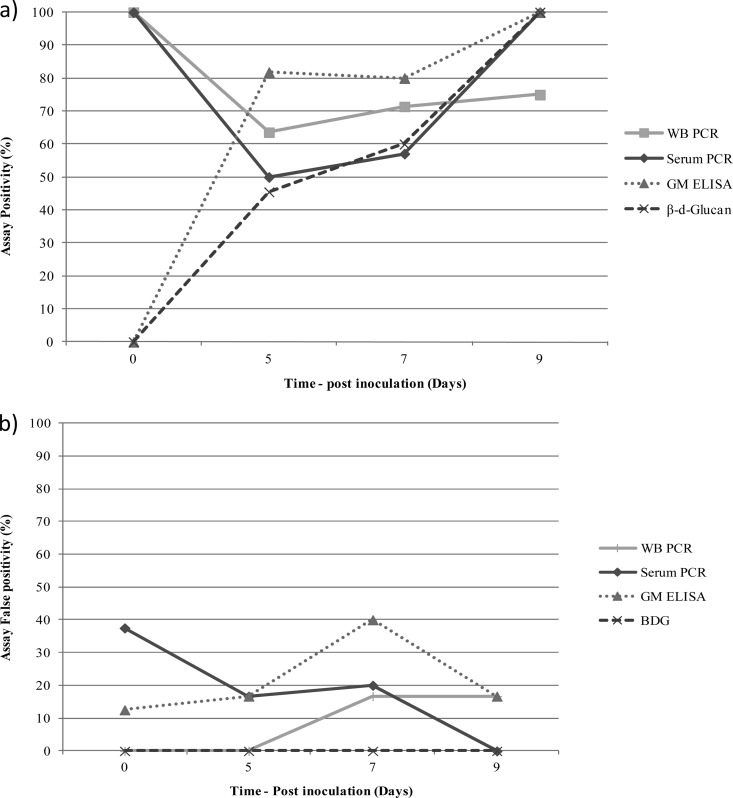
Galactomannan, β-d-glucan, and PCR positivity for the individual time points across the time course of the experiment: (a) infected animals and (b) uninfected animals. Galactomannan positivity was determined using a threshold index of 0.5, and β-d-glucan positivity was determined using a threshold of 80 pg/ml. All PCR positives were confirmed by repeat testing.

**FIG 3:**
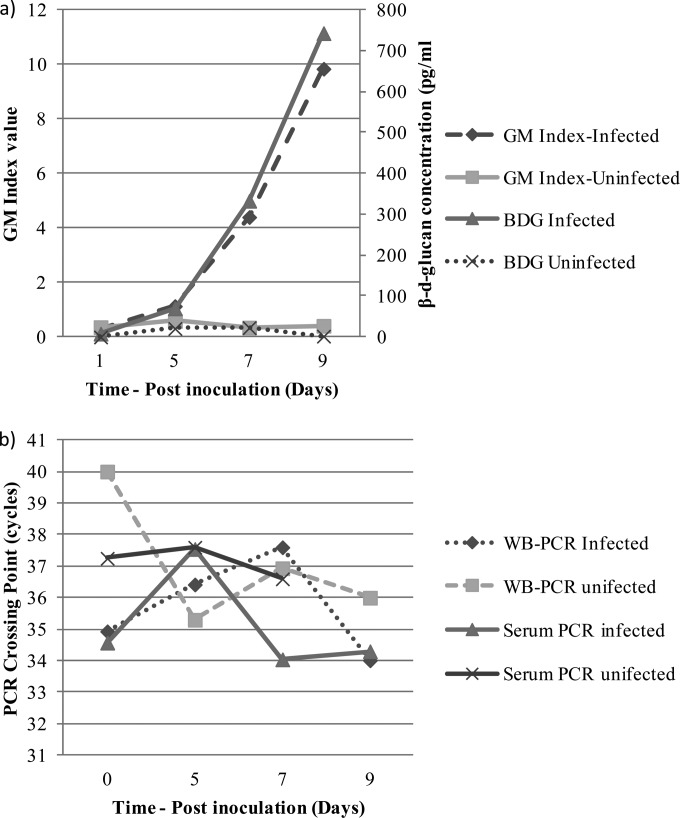
Mean biomarker values over the course of the experiment: (a) galactomannan and β-d-glucan and (b) PCR detection. Values represent the mean figure from infected and uninfected animals at that particular time.

Using the manufacturer's positivity threshold of 80 pg/ml, BDG testing generated excellent specificity, but sensitivity was compromised compared to the other assays (Table 1). The specificity of the BDG assay was significantly superior to that of serum PCR (*P* = 0.0223) and GM testing (*P* = 0.05). ROC analysis showed that to attain sensitivity comparable to the other assays used in this study, the threshold would need to be reduced to 42.73 pg/ml providing sensitivity and specificity values of 63.6% (95% CI, 40.7% to 82.8%) and 92.0% (95% CI, 74.0% to 99.0%), respectively (Fig. 1a). The highest sensitivity (54.6%) while maintaining a specificity of 100% was achieved using a threshold of 58.63 pg/ml. Positivity in infected animals was first seen on day 5 at a positivity rate of 45.5%, and this increased to 60% and 100% on days 7 and 9, respectively (Fig. 2). On day 7, no animals were negative by BDG testing, but two animals were deemed indeterminate generating BDG concentrations of 61.1 and 66.4 pg/ml. The concentration of BDG detected in the serum of infected animals increased during the course of the experiment, with a day 1 mean value of <31.25 pg/ml and rising to 70.0, 333.1, and 742.9 pg/ml on days 5, 7, and 9, respectively (Fig. 3a). The mean concentration of BDG in the serum of uninfected animals over the course of the experiment was <31.25 pg/ml.

### Galactomannan performance.

ROC analysis confirmed that optimal performance was attained using an index of 0.5, although 100% sensitivity or 100% specificity was only achieved using indices of 0.1 and 2.2, respectively, with obvious deleterious effects on diagnostic performance (Fig. 1b). Again, positivity in infected animals was first seen on day 5 at a positivity of 81.8% increasing to 100% by day 9 (Fig. 2a). Unlike BDG testing, GM false positivity was detected throughout the course of the study (rate, 12.5% to 40.0%) (Fig. 2b). Increasing the positivity index to 0.8 reduced the false positives by 80% and generated sensitivity and specificity values of 50.0% and 96.0%, respectively. As with BDG, GM indices increased during the experiment, with a day 1 mean index of 0.3 rising to 1.1, 4.4, and 9.8 on days 5, 7, and 9, respectively (Fig. 3a). The mean index in the serum of uninfected animals over the course of the experiment was 0.4.

### PCR performance.

Overall sensitivity was comparable between the PCR assays and GM testing, although there was a trend toward increased WB PCR sensitivity compared to that of BDG (*P* = 0.0765) (Table 1). Unlike antigen testing, PCR assays were able to detect Aspergillus biomarkers in WB and serum on the day of inoculation, with a slight reduction in positivity on day 5 before increasing during the latter stages of the experiment (Fig. 2a). PCR false positivity was comparable with that for GM EIA (Fig. 2b). Unlike GM and BDG testing, there did not appear to be an increase in available target as disease progressed, and for serum and WB PCR, the relative quantification cycle (*C_q_*) (or threshold cycle) did not increase during the experiment (Fig. 3b). Indeed, *C_q_* values were similar in infected and uninfected animals (for WB PCR, 35.7 versus 36.8 cycles), although the frequency of PCR positivity was significantly greater in infected animals. For WB PCR, the difference in PCR positivity rates across the duration of the study in infected compared to the false positivity rate in uninfected animals was 51.8% (*P* < 0.0001), and for serum PCR the difference was 46.0% (*P* < 0.0001).

### Combination testing.

In this study, BDG testing alone provided 100% specificity across the duration of the study, but sensitivity was compromised (45.5%) resulting in a poor likelihood ratio for a negative test result (LR−, 0.55) (Table 1). When combining two biomarker assays, GM and WB PCR improved sensitivity to 100% if one of the two assays was positive, representing significant increases in sensitivity of 27% (*P* = 0.0123) and 32% (*P* = 0.0089) compared to the individual use of WB PCR and GM, respectively. Using this dual WB PCR/GM strategy, there was no significant reduction in specificity compared to that of individual use of the assays (GM, *P* = 1.00; WB PCR, *P* = 0.23), and >95% specificity was attained if the two assays were positive (Table 1). No dual assay strategy was able to confidently exclude and diagnose IA (i.e., 100% sensitivity and specificity), and a minimum combination of three assays was required to achieve this goal. Combining either WB or serum PCR with the two antigen tests or combining the two PCR tests with GM provided performance such that if all assays were positive an animal could be diagnosed with IA, whereas if all assays were negative disease could be excluded (Table 1). Of the triple strategies, the WB PCR/GM/BDG approach using a threshold of a single positive assay as significant provided the best diagnostic accuracy (89%). It was confirmed by ROC analysis, where the combination of WB PCR/BDG/GM provided an AUC that was superior to the other triplet strategies and that was comparable to the AUC generated when combining all four assays, that the latter strategy provided no additional benefit over using WB PCR in combination with the two antigen tests.

## DISCUSSION

In this study, the performance of standardized PCR methodologies proposed by the EAPCRI for testing serum and WB was compared to that of antigen detection in an animal model of IA. BDG testing provided excellent specificity, but sensitivity was compromised until the late stages of the experiment. Unlike the clinical scenario, the animals in this study were not exposed to the various clinical factors associated with false-positive BDG results (14). Consequently, the specificity of the BDG assay for the detection of IA may be higher than that seen in the clinic and may be further compounded by the ability to detect but not differentiate fungal pathogens other than Aspergillus. In this model, the optimal positivity threshold was 58.6 pg/ml; however, given the limitations stated above, in clinical practice it would be wise not to lower the current threshold. For GM, the current positivity index of 0.5 provided optimal overall performance (sensitivity, 68.2% [95% CI, 45.1% to 86.1%]; specificity, 84.0% [95% CI, 63.9% to 95.5%]) with an index of 1.0 generating a specificity of 96.0% (95% CI, 79.7% to 99.9%) and positive likelihood ratio of 12.5, which is sufficient to confirm disease.

Disease progression coincided with increasing levels of GM and BDG, whereas PCR testing performed better during early stages. Serum and WB PCR assays were uniformly positive 1 h after inoculation of the animals, a period that precludes the disease process, as the formation of hyphae, required for invasion, takes between 6 and 24 h to develop (15). This suggests that PCR positivity in blood can reflect exposure and is not solely dependent on angioinvasion. This probably represents detection of phagocytosed conidia within translocated alveolar macrophages (16) and may provide an opportunity to initiate targeted prophylaxis or preemptive therapy to preempt disease in at-risk patients who have had a significant exposure. Given the limited number of animals tested at this time point, further testing is warranted to confirm the significance of this finding.

False positivity was noted for GM and the two PCR assays but was significantly less than the corresponding true positivity rates. Comparing the immunosuppressed control population with the immunocompetent control population, false positivity for GM and PCR was greater in the immunosuppressed population (difference of 15.0% [95% CI, 0.9% to 28.5%]; *P* = 0.043). It is possible that this represented natural infection and slower clearance of conidia inhaled from the environment. No false positivity was noted with BDG testing, and this may simply be the result of reduced sensitivity or because the animals had not been exposed to agents associated with false positivity in the clinic.

The study gives new insights into the timing of biomarker release critical to their use as a test in a preemptive or diagnostic-driven approach. GM and BDG are released during active hyphal growth, which is indicative of progression of infection to disease (17). Cordonnier et al. showed that a diagnostic approach based on radiological and GM testing increased the incidence of IA diagnosis with no overall effect on mortality rates, although antifungal usage was reduced (18). Targeting GM and BDG alone may detect early infection in a diagnostic-driven strategy but may be insufficient to preempt disease. In this study, PCR positivity predated antigen positivity, and this has been shown in the clinical setting, where in one study DNAemia was detected on average 68 days before GM and in a second where PCR preceded GM in 15 patients with IA (19–21). Not all susceptible patients exposed to Aspergillus will develop disease, and PCR will generate false-positive results with respect to IA. However, this information may be used to direct prophylactic and preemptive strategies, particularly in patients at increased risk of IA. In particular, PCR testing can be combined with genetic screening of the host to determine mutations in immunity, such as dectin-1, which increases susceptibility, and further support initiating preemptive therapy (22).

However, the timing of presentation of the patient is critical, and antigen testing may prove beneficial for a symptomatic patient presenting with clinical signs typical of IA without any prior diagnostic work-up, particularly as, in this study, antigen levels increased during the course of the experiment. A combined diagnostic strategy can be used to preempt, diagnose, and monitor disease progression, in addition to excluding disease. Millon et al. performed real-time PCR on the first GM-positive serum, and patients positive by PCR and GM had a poor prognosis (23). In diagnosing the disease, this strategy is adequate; however, for screening or preemptive strategies, it may be beneficial to reverse this algorithm using PCR to detect possible exposure/early infection and antigen testing to monitor for disease progression. By combining multiple biomarker tests, IA can be confidently excluded or confirmed. This result highlights the need for a diagnostic strategy using combination testing to screen or, alternatively, to confirm diagnosis. Four tests were evaluated in this study. Combining either of the PCR assays with BDG and GM or both PCR assays with GM allowed disease to be excluded or diagnosed. This approach must be balanced against the practicality and cost of using multiple tests. Recent clinical trials, supported by a meta-analysis, comparing a combination approach using GM and Aspergillus PCR with conventional methods, have shown significant benefits, including a reduction in disease burden (24–26). While the cost of multiple tests may seem prohibitive, it can be more than offset by the reduction in unnecessary antifungal therapy driven by the high sensitivity and negative predictive value of combination testing (24–26). From a practical standpoint, serum/plasma PCR and GM can be performed on the same sample and can be automated, reducing pressure on the laboratory (27). WB and serum PCR can be streamlined by combining serum and WB DNA extraction methods into a single process resulting in a single PCR test to be combined with GM (28). Unfortunately, due to limited sample material, it was not possible to evaluate this combined approach in this study. Another limitation is that plasma was not available, as all EDTA WB was frozen post sampling. In human material, PCR and GM testing of plasma has been shown to generate superior results over serum, and it would have been beneficial if this were investigated in this study (8, 29, 30).

These results confirm satisfactory performance of the EAPCRI recommendations for PCR testing of serum and WB compared to that of commercially available antigen tests. It is essential that the performance of the EAPCRI recommendations is fully evaluated in a clinical trial to determine accurate clinical performance.

## References

[1] De Pauw B, Walsh TJ, Donnelly JP, Stevens DA, Edwards JE, Calandra T, Pappas PG, Maertens J, Lortholary O, Kauffman CA, Denning DW, Patterson TF, Maschmeyer G, Bille J, Dismukes WE, Herbrecht R, Hope WW, Kibbler CC, Kullberg BJ, Marr KA, Muñoz P, Odds FC, Perfect JR, Restrepo A, Ruhnke M, Segal BH, Sobel JD, Sorrell TC, Viscoli C, Wingard JR, Zaoutis T, Bennett JE, European Organization for Research and Treatment of Cancer/Invasive Fungal Infections Cooperative Group, National Institute of Allergy and Infectious Diseases Mycoses Study Group (EORTC/MSG) Consensus Group. 2008 Revised definitions of invasive fungal disease from the European Organization for Research and Treatment of Cancer/Invasive Fungal Infections Cooperative Group and the National Institute of Allergy and Infectious Diseases Mycoses Study Group (EORTC/MSG) Consensus Group. Clin Infect Dis 46:1813–1821. doi:10.1086/588660. 18462102 PMC2671227

[2] White PL, Bretagne S, Klingspor L, Melchers WJG, McCulloch E, Schulz B, Finnstrom N, Mengoli C, Barnes RA, Donnelly JP, Loeffler J, European Aspergillus PCR Initiative. 2010 Aspergillus PCR: one step closer towards standardization. J Clin Microbiol 48:1231–1240. doi:10.1128/JCM.01767-09. 20147637 PMC2849576

[3] White PL, Mengoli C, Bretagne S, Cuenca-Estrella M, Finnstrom N, Klingspor L, Melchers WJ, McCulloch E, Barnes RA, Donnelly JP, Loeffler J, European Aspergillus PCR Initiative (EAPCRI). 2011 Evaluation of Aspergillus PCR protocols for testing serum specimens. J Clin Microbiol 49:3842–3848. doi:10.1128/JCM.05316-11. 21940479 PMC3209112

[4] Lengerova M, Kocmanova I, Racil Z, Hrncirova K, Pospisilova S, Mayer J, Najvar LK, Wiederhold NP, Kirkpatrick WR, Patterson TF 2012 Detection and measurement of fungal burden in a guinea pig model of invasive pulmonary aspergillosis by novel quantitative nested real-time PCR compared with galactomannan and (1,3)-β-d-glucan detection. J Clin Microbiol 50:602–608. doi:10.1128/JCM.05356-11. 22189110 PMC3295131

[5] Kirkpatrick WR, Najvar LK, Vallor AC, Wiederhold NP, Bocanegra R, Pfeiffer J, Perkins K, Kugler AR, Sweeney TD, Patterson TF 2012 Prophylactic efficacy of single dose pulmonary administration of amphotericin B inhalation powder in a guinea pig model of invasive pulmonary aspergillosis. J Antimicrob Chemother 67:970–976. doi:10.1093/jac/dkr567. 22240402

[6] White PL, Perry MD, Barnes RA 2009 Polymerase chain reaction diagnosis of fungal disease: finally coming of age. Curr Fungal Infect Rep 3:207–215. doi:10.1007/s12281-009-0029-3.

[7] Springer J, Morton CO, Perry M, Heinz WJ, Paholcsek M, Alzheimer M, Rogers TR, Barnes RA, Einsele H, Loeffler J, White PL 2013 Multicenter comparison of serum and whole-blood specimens for detection of Aspergillus DNA in high-risk hematological patients. J Clin Microbiol 51:1445–1450. doi:10.1128/JCM.03322-12. 23426930 PMC3647891

[8] White PL, Barnes RA, Springer J, Klingspor L, Cuenca-Estrella M, Morton CO, Lagrou K, Bretagne S, Melchers WJG, Mengoli C, Donnelly JP, Heinz WJ, Loeffler J 2015 Clinical performance of Aspergillus PCR for testing serum and plasma: a study by the European Aspergillus PCR Initiative. J Clin Microbiol 53:2832–2837. doi:10.1128/JCM.00905-15. 26085618 PMC4540904

[9] National Academy of Sciences. 1996 Guide for the care and use of laboratory animals. National Academy Press, Washington, DC.

[10] Graybill JR, Kaster SR 1984 Experimental murine aspergillosis. Comparison of amphotericin B and a new polyene antifungal drug, SCH 28191. Am Rev Respir Dis 129:292–295. 6364905

[11] White PL, Linton CJ, Perry MD, Johnson EM, Barnes RA 2006 The evolution and evaluation of a whole blood polymerase chain reaction assay for the detection of invasive aspergillosis in hematology patients in a routine clinical setting. Clin Infect Dis 42:479–486. doi:10.1086/499949. 16421791

[12] Millon L, Grenouillet F, Legrand F, Loewert S, Bellanger AP, Gbaguidi-Haore H, Scherer E, Henon T, Rohrlich P, Deconinck E 2011 Ribosomal and mitochondrial DNA target for real-time PCR diagnosis of invasive aspergillosis. J Clin Microbiol 49:1058–1063. doi:10.1128/JCM.01904-10. 21227993 PMC3067699

[13] Newcombe RG 1998 Interval estimation for the difference between independent proportions: comparison of eleven methods. Stat Med 17:873–890. doi:10.1002/(SICI)1097-0258(19980430)17:8<873::AID-SIM779>3.0.CO;2-I. 9595617

[14] White PL, Wingard JR, Bretagne S, Löffler J, Patterson TF, Slavin MA, Barnes RA, Pappas PG, Donnelly JP 2015 Aspergillus polymerase chain reaction: systematic review of evidence for clinical use in comparison with antigen testing. Clin Infect Dis 61:1293–1303. doi:10.1093/cid/civ507. 26113653 PMC4583581

[15] Morton CO, Loeffler J, De Luca A, Frost S, Kenny C, Duval S, Romani L, Rogers TR 2010 Dynamics of extracellular release of Aspergillus fumigatus DNA and galactomannan during growth in blood and serum. J Med Microbiol 59:408–413. doi:10.1099/jmm.0.017418-0. 20035025

[16] Furuyama A, Kanno S, Kobayashi T, Hirano S 2009 Extrapulmonary translocation of intratracheally instilled fine and ultrafine particles via direct and alveolar macrophage-associated routes. Arch Toxicol 83:429–437. doi:10.1007/s00204-008-0371-1. 18953527

[17] Mennink-Kersten MA, Ruegebrink D, Wasei N, Melchers WJ, Verweij PE 2006 *In vitro* release by Aspergillus fumigatus of galactofuranose antigens, 1,3-β-d-glucan, and DNA, surrogate markers used for diagnosis of invasive aspergillosis. J Clin Microbiol 44:1711–1718. doi:10.1128/JCM.44.5.1711-1718.2006. 16672397 PMC1479172

[18] Cordonnier C, Pautas C, Maury S, Vekhoff A, Farhat H, Suarez F, Dhédin N, Isnard F, Ades L, Kuhnowski F, Foulet F, Kuentz M, Maison P, Bretagne S, Schwarzinger M 2009 Empirical versus preemptive antifungal therapy for high-risk, febrile, neutropenic patients: a randomized, controlled trial. Clin Infect Dis 48:1042–1051. doi:10.1086/597395. 19281327

[19] Meije Y, Aguado JM, Cuenca-Estrella M 2011 Silent and prolonged Aspergillus DNAemia in oncohematological patients receiving antifungal prophylaxis: a new phenomenon with clinical implications. Bone Marrow Transplant 46:1016–1017. doi:10.1038/bmt.2010.219. 20871641

[20] Barnes RA, White PL, Bygrave C, Evans N, Healy B, Kell J 2009 Clinical impact of enhanced diagnosis of invasive fungal disease in high-risk haematology and stem cell transplant patients. J Clin Pathol 62:64–69. doi:10.1136/jcp.2008.058354. 19103864

[21] Barnes RA, Stocking K, Bowden S, Poynton MH, White PL 2013 Prevention and diagnosis of invasive fungal disease in high-risk patients within an integrative care pathway. J Infect 67:206–214. doi:10.1016/j.jinf.2013.04.020. 23644098

[22] Hardison SE, Brown GD 2012 C-type lectin receptors orchestrate antifungal immunity. Nat Immunol 13:817–822. doi:10.1038/ni.2369. 22910394 PMC3432564

[23] Millon L, Piarroux R, Deconinck E, Bulabois CE, Grenouillet F, Rohrlich P, Costa JM, Bretagne S 2005 Use of real-time PCR to process the first galactomannan-positive serum sample in diagnosing invasive aspergillosis. J Clin Microbiol 43:5097–5101. doi:10.1128/JCM.43.10.5097-5101.2005. 16207969 PMC1248437

[24] Aguado JM, Vázquez L, Fernandez-Ruiz M, Villaescusa T, Ruiz-Camps I, Barba P, Silva JT, Batlle M, Solano C, Gallardo D, Heras I, Polo M, Varela R, Vallejo C, Olave T, Lopez-Jimenez J, Rovira M, Parody R, Cuenca-Estrella M, PCRAGA Study Group, Spanish Stem Cell Transplantation Group, Study Group of Medical Mycology of the Spanish Society of Clinical Microbiology and Infectious Diseases, Spanish Network for Research in Infectious Diseases. 2015 Serum galactomannan versus a combination of galactomannan and PCR-based Aspergillus DNA detection for early therapy of invasive aspergillosis in high-risk hematological patients: a randomized controlled trial. Clin Infect Dis 60:405–414. doi:10.1093/cid/ciu833. 25336623

[25] Morrissey CO, Chen SC, Sorrell TC, Milliken S, Bardy PG, Bradstock KF, Szer J, Halliday CL, Gilroy NM, Moore J, Shcwarer AP, Guy S, Bajel A, Tramontana AR, Spelman T, Slavin MA, Australasian Leukaemia Lymphoma Group and the Australia and New Zealand Mycology Interest Group. 2013 Galactomannan and PCR versus culture and histology for directing use of antifungal treatment for invasive aspergillosis in high-risk haematology patients: a randomised controlled trial. Lancet Infect Dis 13:519–528. doi:10.1016/S1473-3099(13)70076-8. 23639612

[26] Arvanitis M, Anagnostou T, Mylonakis E 2015 Galactomannan and polymerase chain reaction-based screening for invasive aspergillosis among high-risk hematology patients: a diagnostic meta-analysis. Clin Infect Dis 61:1263–1272. doi:10.1093/cid/civ555. 26157047

[27] Gorton RL, White PL, Bagkeris E, Cotterall D, Desai R, McHugh T, Kibbler CC 2015 Improved standardization of the Bio-Rad Platelia Aspergillus galactomannan antigen sandwich enzyme immunoassay using the DS2 (Dynex) enzyme-linked immunosorbent assay (ELISA) processing system. J Clin Microbiol 53:2072–2078. doi:10.1128/JCM.00157-15. 25878352 PMC4473217

[28] Springer J, Schloßnagel H, Heinz W, Doedt T, Soeller R, Einsele H, Loeffler J 2012 A novel extraction method combining plasma with a whole-blood fraction shows excellent sensitivity and reproducibility for patients at high risk for invasive aspergillosis. J Clin Microbiol 50:2585–2591. doi:10.1128/JCM.00523-12. 22593600 PMC3421527

[29] White PL, Jones T, Whittle K, Watkins J, Barnes RA 2013 Comparison of galactomannan enzyme immunoassay performance levels when testing serum and plasma samples. Clin Vaccine Immunol 20:636–638. doi:10.1128/CVI.00730-12. 23389933 PMC3623416

[30] Loeffler J, Mengoli C, Springer J, Bretagne S, Cuenca Estrella M, Klingspor L, Lagrou K, Lass-Floerl C, Melchers W, Millon L, Morton O, Barnes RA, Donnelly JP, White PL, European Aspergillus Initiative PCR. 2015 Analytical comparison of *in vitro* spiked human serum and plasma for PCR-based detection of Aspergillus fumigatus DNA: a study by the European Aspergillus PCR Initiative. J Clin Microbiol 53:2838–2845. doi:10.1128/JCM.00906-15. 26085614 PMC4540929

